# Supplementary material for: Content-rich biological network constructed by mining PubMed abstracts
Source: BMC Bioinformatics. 2004 Oct 8;5:147. doi: 10.1186/1471-2105-5-147 (PMC528731; doi:10.1186/1471-2105-5-147)
Supplement: Additional File 2 — The original results of the above study (non-essential files are deleted to keep the file size under the limit set by BMC bioinformatics). [file 1471-2105-5-147-S2.bz2 › chilibotAdditionalFile2/dip05/293ID7033218E1125/html/NADB_NADA.html]

 


 **NADB** and **NADA** 
  
Found 14 abstracts in PubMed, retrieved 05.  
 

 What does Google say? 
 PDF only 
| .edu only 

---

**Interactive relationship** (e.g. stimulation, inhibition, etc)

**Neutral relationship**- In several bacteria, the gene in the position occupied by TM1643 encodes an aspartate oxidase  **NadB** , which synthesizes iminoaspartate as a substrate for  **NadA** , the next enzyme in the pathway.  Ref: 12496312 J Biol Chem, 2003
- Quinolinic acid is synthesized in E. coli by the enzymes L aspartate oxidase  [ **NADB** ]  and quinolinate synthase A, the genes of which are named  **nadB**  and  **nadA** .  Ref: 2187483 Biol Chem Hoppe Seyler, 1990

**Non-interactive relationship** (e.g. studied together, co-existance, homology, etc.)

- As NAD levels increase within the cell, the affinity of NadR for the operator regions of  **nadA** ,  **nadB** , and pncB increases, repressing the transcription of these target genes.  Ref: 2198247 J Bacteriol, 1990
- In Salmonella typhimurium, de novo synthesis of NAD is regulated through the transcriptional control of the  **nadA**  and  **nadB**  loci.  Ref: 2198247 J Bacteriol, 1990
- Finally, our data definitely exclude the possibility that other enzymes are involved in the biosynthesis of quinolinic acid in E. coli, since it is possible to synthesize quinolinic acid from l aspartate, dihydroxyacetone phosphate, and O 2 by using only  **nadA**  and  **nadB**  gene overexpressed products.  Ref: 10648170 Protein Expr Purif, 2000
- The nadI protein regulates transcription of the  **nadA**  and  **nadB**  biosynthetic genes and modulates activity of the NMN permease.  Ref: 1991724 J Bacteriol, 1991
